# Supplementary material for: O-GlcNAcylation of YTHDF2 antagonizes ERK-dependent phosphorylation and inhibits lung carcinoma
Source: Fundam Res. 2024 Jul 26;5(5):2388–96. doi: 10.1016/j.fmre.2024.07.003 (PMC12848141; doi:10.1016/j.fmre.2024.07.003)
Supplement: Supplementary file 1 [file mmc1.pdf]

Supplemental Figure 1. YTHDF2 O-GlcNAcylation in H1299 cells.

A, H1299 cells were transfected with HA-YTHDF2-WT or -T49A plasmids, treated or untreated with the OGA inhibitor Thiamet-G (TMG) and glucose. Then the anti-HA immunoprecipitates were immunoblotted with RL2 antibodies. B -E, Cells were transfected with HA-YTHDF2-WT or HA-YTHDF2-S39A (B), or -T49A (D) plasmids, and then the lysates were immunoprecipitated with anti-HA antibodies and immunoblotted with indicated antibodies. C Quantitation of (B). E Quantitation of (D). F-I, Cells were transfected with Flag-OGT together with HA-YTHDF2-WT or -S39A (F), or Flag-Erk together with HA-YTHDF2-WT and -T49A (H), and then the extracts were subject to immunoprecipitation with anti-Flag antibodies and immunoblotted with the indicated antibodies. G, Quantitation of (F). I, Quantitation of (H). J, cells were transfected with HA-YTHDF2-WT or -T49A plasmids and the lysates were immunoprecipitated with anti-HA antibodies and immunoblotted with anti-ubiquitin antibodies. K. Quantitation of (J). L, cells were transfected with HA-YTHDF2-WT and Flag-OGA plasmids and the lysates were immunoprecipitated with anti-HA antibodies and immunoblotted with anti-ubiquitin antibodies. M, Quantitation of (L). N-O, Cycloheximide (CHX) pulse-chase assays. H1299 cells were transfected with HA-YTHDF2-WT or -T49A plasmids, treated with CHX, and then collected at different time points. Cell extracts were blotted with anti-HA and anti-actin antibodies. O. The quantitation of (O=N). * indicates $P < 0.05$, **** indicates $p<0.0001$. All Western blots were repeated for at least three times.
