# Supplementary figures and images for: O-GlcNAcylation of YTHDF2 antagonizes ERK-dependent phosphorylation and inhibits lung carcinoma
Source: Fundam Res. 2024 Jul 26;5(5):2388–96. doi: 10.1016/j.fmre.2024.07.003 (PMC12848141; doi:10.1016/j.fmre.2024.07.003)

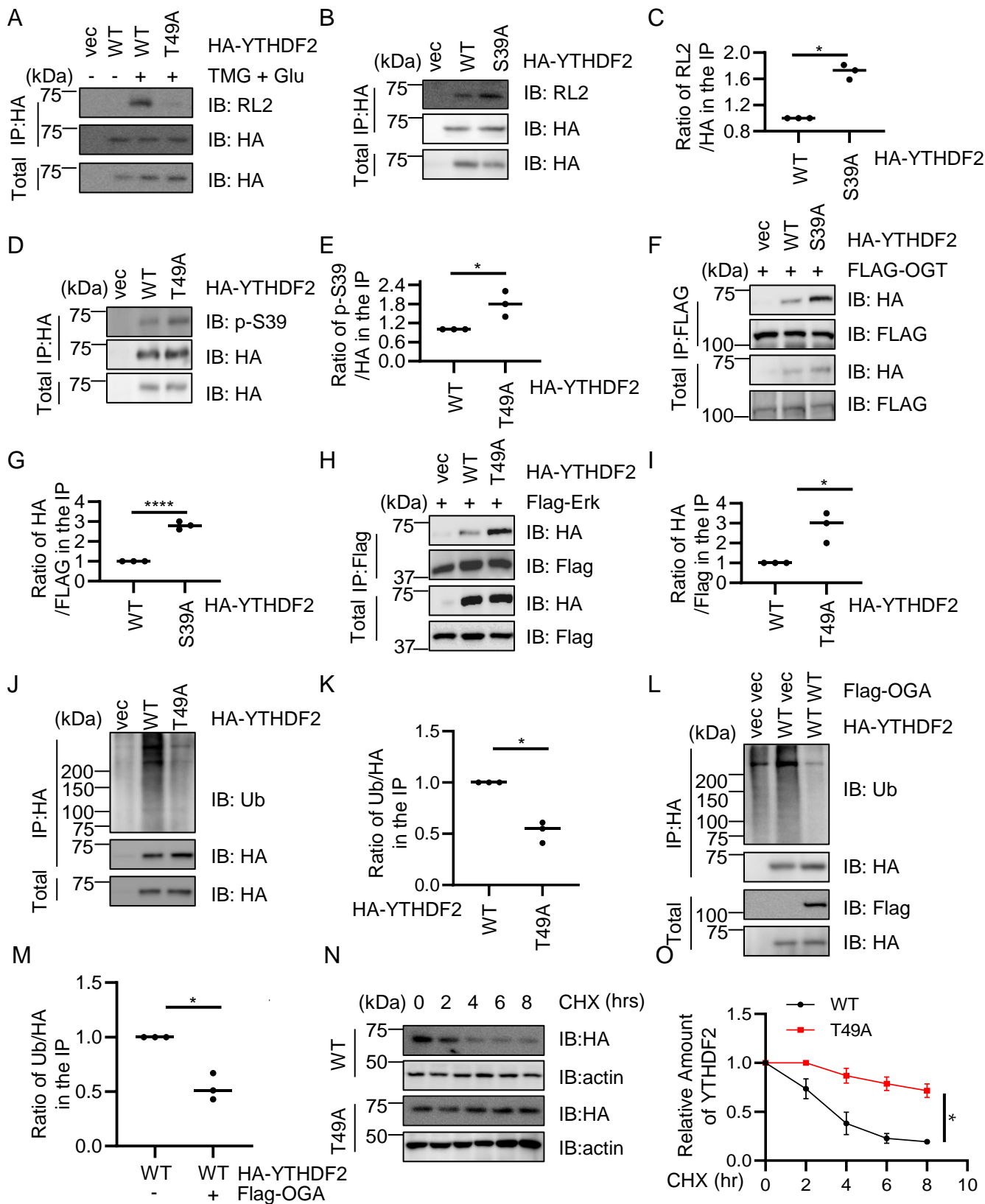

Supplementary Figure 1

Supplement: Supplementary file 2 [file mmc2.pdf]
